# Assessing Melt Flow Rate in Post-Consumer Polypropylene via Near-Infrared Hyperspectral Imaging

**DOI:** 10.3390/polym18040524

**Published:** 2026-02-20

**Authors:** Nikolai Kuhn, Moritz Mager, Gerald Koinig, Jutta Geier, Jean-Philippe Andreu, Joerg Fischer, Alexia Tischberger-Aldrian

**Affiliations:** 1 Chair of Waste Processing Technology and Waste Management, Department of Environmental and Energy Process Engineering, Technical University of Leoben, 8700 Leoben, Austria; 2 Institute of Polymeric Materials and Testing, Johannes Kepler University Linz, 4040 Linz, Austria; 3 LIT Factory, Johannes Kepler University Linz, 4040 Linz, Austria; 4 Polymer Competence Center Leoben GmbH, 8700 Leoben, Austria; 5 Chair of Materials Science and Testing of Polymers, Department for Polymer Engineering and Science, Technical University of Leoben, 8700 Leoben, Austria; 6 Intelligent Vision Applications, Joanneum Research Forschungsgesellschaft mbH, 8010 Graz, Austria

**Keywords:** plastic recycling, mechanical recycling, sensor-based sorting, sensor-based monitoring and control

## Abstract

Mechanical recycling of polypropylene (PP) is constrained by the heterogeneous properties of post-consumer feedstocks. Melt flow rate (MFR) is a key property relevant to processing, and it varies widely across packaging grades, which limits the quality and substitutability of recyclates. This study evaluates near-infrared hyperspectral imaging (NIR-HSI) for predicting MFR in post-consumer PP packaging. Eighty-two rigid PP samples (46 white, 36 clear) with MFR values between 2 and 108 g 10 min^−1^ were collected from an Austrian material recovery facility. Thirteen different linear and non-linear regression models were examined using median and pixel-wise aggregated spectral representations across the samples. Tree-based models consistently achieved best performances with R^2^ = 0.85, RMSE = 12.4 g 10 min^−1^ on white samples and R^2^ = 0.61, RMSE = 14.0 g 10 min^−1^ on clear samples. On the combined sample set, R^2^ = 0.66 and RMSE = 17.3 g 10 min^−1^ were reached. Informative spectral regions correspond to typical bands of PP. Binary classification at different thresholds (6, 12, 30, 60 g 10 min^−1^) were also examined and achieved balanced accuracies of 0.82–0.92. Median spectral representations consistently outperformed pixel-wise aggregation. Results demonstrate that NIR-HSI can support grade-directed sorting of post-consumer PP, particularly for opaque white samples, though heteroscedasticity at high MFR values and irreducible outliers represent inherent limitations.

## 1. Introduction

Plastics are widespread across sectors, with packaging alone accounting for approximately 40% of total plastic use [1]. Recognizing the environmental imperative to close material loops, the European Union’s Packaging and Packaging Waste Regulation (PPWR) mandates minimum recycled-content targets by 2030—for instance, 10% for contact-sensitive packaging made from plastics other than polyethylene terephthalate (PET) and 35% for non-contact-sensitive packaging [2]. While these requirements are expected to stimulate demand for high-quality recyclates, regulatory pull alone does not overcome the technical limitations constraining mechanical recycling.

These limitations arise from multiple sources: First, thermo-mechanical stress during reprocessing and cumulative degradation over product lifetimes reduce polymer chain length and alter rheological behavior [3,4,5,6]. Second, foreign materials from incomplete cleaning and sorting introduce contamination that further compromises material quality [5,7,8,9]. Third, and most fundamentally, virgin polymers are manufactured in a wide diversity of grades tailored to application-specific requirements [10,11,12]. At end of life, however, post-consumer packaging is typically collected and processed together, separated only by polymer type. The resulting recyclates therefore exhibit blended, averaged properties [13,14], and the application-specific characteristics of virgin grades are lost—reducing the extent to which recyclates can substitute virgin materials [15,16,17,18].

Sensor-based sorting offers a pathway to improve recyclate quality. Deployed both at the article level in material recovery facilities and at the flake level after wet-mechanical treatment, sensor-based systems detect particles and mechanically separate them according to measured properties [19,20]. Beyond sorting, the same sensors can be leveraged for in-line material-flow characterization, enabling adaptive process control [21]. Among available sensing modalities, hyperspectral imaging (HSI) is particularly attractive because it is not only contactless and real-time capable, but it also combines broad spectral coverage with high spectral and spatial resolution at low noise [22,23]. Thus, HSI provides access to vibrational spectroscopy and is thereby employed to detect characteristic molecular absorption bands [24,25,26,27]. Although the mid-infrared (MIR, 2500–25,000 nm) contains fundamental vibrational modes, the near-infrared (NIR, 780–2500 nm) is more practical for industrial deployment despite its lower-intensity overtones and combination bands [26]. Industrial practice has consequently focused on NIR-HSI for polymer-type discrimination [28,29,30]. Recent studies, however, suggest that NIR-HSI may also capture chemical and processing-related properties: aging and degradation in polyethylene (PE) and PP [31,32], density and MFR in PE [33], multilayer-film composition [34,35,36], and density, crystallinity, and short-chain branching in PE [37].

Among commodity polymers, PP merits particular attention. Representing approximately 19% of global plastics production [38], PP owes its broad utility to a low-cost monomer, a low glass-transition temperature, and tunability via comonomer content and molar mass [39,40]. PP is processed by extrusion, blow molding, and injection molding, and commercial packaging grades span a wide MFR range of approximately 1.3–100 g 10 min^−1^ [10]. MFR, defined as the mass of polymer extruded in ten minutes under standardized conditions (ISO 1133-1:2022) [41], is a key property relevant to processing governed primarily by molar mass [42] but also molar mass distribution [42], and branching [43]. Notably, several of these factors may also manifest in NIR spectra, presenting both opportunities and challenges for property-based sorting.

Despite this potential, studies on NIR-HSI prediction of technical properties in post-consumer PP—particularly MFR—remain scarce. Reference [44] sorted post-consumer PP by opacity and translucency and achieved reduced MFR variability, yet concluded that direct sorting by polymer-grade characteristics would be more robust. Reference [45] examined nine clear PP packaging items, recording NIR spectra and measuring tensile strength and melt viscosity. Partial least squares (PLS) regression yielded strong correlations for tensile strength (R^2^ = 0.95) and dynamic viscosity (R^2^ = 0.97); however, distinguishing packages with the same function across brands proved challenging, and the small sample size, narrow viscosity range (930–7317 Pa·s), and focus on clear, off-the-shelf packaging limit generalizability. Overall, a systematic evaluation of NIR-HSI for predicting PP MFR across realistic post-consumer streams remains lacking.

This study aims to assess whether NIR-HSI can predict the MFR of post-consumer PP packaging with an accuracy that is sufficient for grade-directed sorting. We provide a systematic evaluation across translucent and opaque samples, compare object-wise (median spectrum) and pixel-wise modeling strategies, and identify the spectral regions most informative for MFR prediction. Specifically, we address the following research questions (RQ):

- RQ 1: Is there a detectable spectral relationship between NIR-HSI spectra and MFR in post-consumer PP?
- RQ 2: Which spectral bands carry the most predictive information for MFR?
- RQ 3: How does prediction performance vary with sample translucency/opacity and with object-wise versus pixel-wise modeling?
- RQ 4: Is the relationship better suited to regression or classification?

## 2. Materials and Methods

### 2.1. Sample Collection and Preparation

To obtain a realistic sample set, 82 rigid PP packages (36 translucent clear, 46 opaque white) were collected at an Austrian material recovery facility (MRF) handling separately collected post-consumer lightweight packaging waste from Austria and Southern Germany. Samples were picked from the conveyor belt after the final sorting step (sorting fraction: translucent clear and opaque white rigid PP). The selection aimed to cover the principal conversion processes—blow molding (BM), thermoforming (TF), and injection molding (IM)—as well as all major application categories of rigid PP packaging. Colored packaging was excluded to avoid confounding effects from pigments and associated additives, but printed packaging was included.

**Figure 1:**
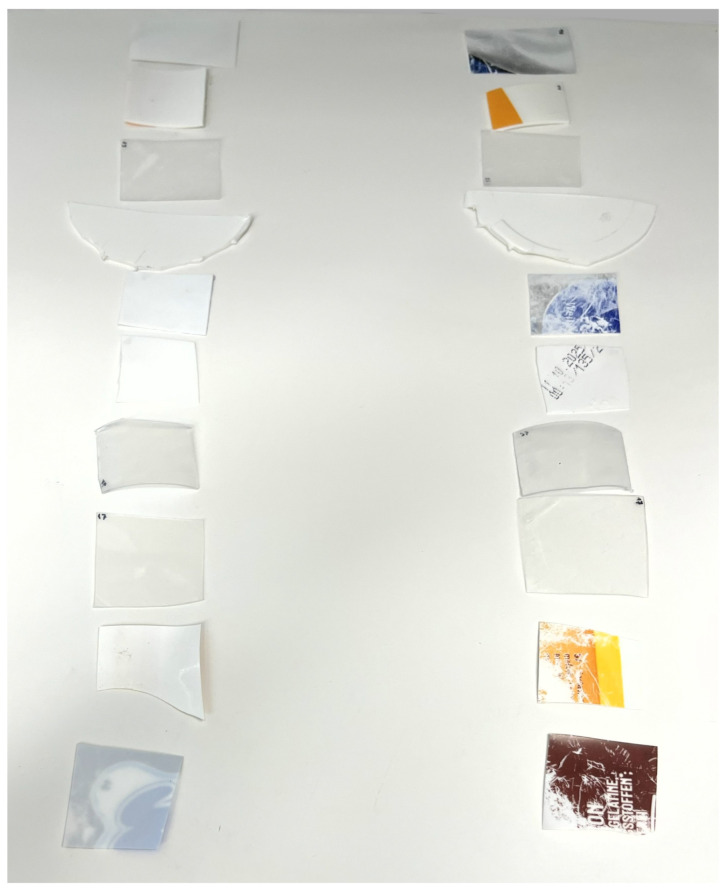
Depiction of 20 samples during spectral acquisition. Each sample stems from a previously collected post-consumer PP lightweight packaging. To avoid interfering spectral artifacts, detachable components were removed and samples were washed beforehand. Printed clear/white packaging was included.

Detachable components (e.g., lids, labels, sealing films) were manually removed, and samples were pre-washed in a domestic washing machine (PW 818, Miele, Gütersloh, Germany) for 5 min at 60 °C in 0.5% (*w*/*v*) sodium hydroxide solution. Each sample was then sectioned: a specimen of at least 20 × 20 mm was reserved for hyperspectral imaging (see Figure 1), while the remainder was retained for melt flow rate (MFR) determination.

MFR was measured using a melt flow indexer (Aflow, ZwickRoell, Ulm, Germany) in accordance with ISO 1133-1:2022, at 230 °C with a 2.16 kg load. Measurements were performed directly on flakes prepared from each sample, with six replicate extrusions per sample. The arithmetic mean of the six replicates was used as the reference MFR value.

Hyperspectral imaging was performed using a push-broom line-scan NIR camera (HAIP BlackIndustry 1.7, HAIP Solutions, Hannover, Germany) covering 952–1748 nm at 2 nm spectral resolution. The camera was mounted in a benchtop configuration at a sample-to-lens distance of 33 cm. Acquisition parameters replicated typical industrial sensor-based sorting conditions: 100 lines per second at 2500 µs integration time. Dark-current and white-reference calibrations were performed immediately before each imaging session.

Regions of interest (ROIs) corresponding to each sample were manually delineated, and pixel spectra within each ROI were extracted. To exclude spectra corrupted by noise or edge effects, the per-wavelength standard deviation was computed across all pixels for each sample; spectra deviating by more than three standard deviations from the sample mean were discarded.

All 82 samples underwent both MFR characterization and hyperspectral imaging as well as modeling.

### 2.2. Experimental Design and Sample Grouping

**Figure 2:**
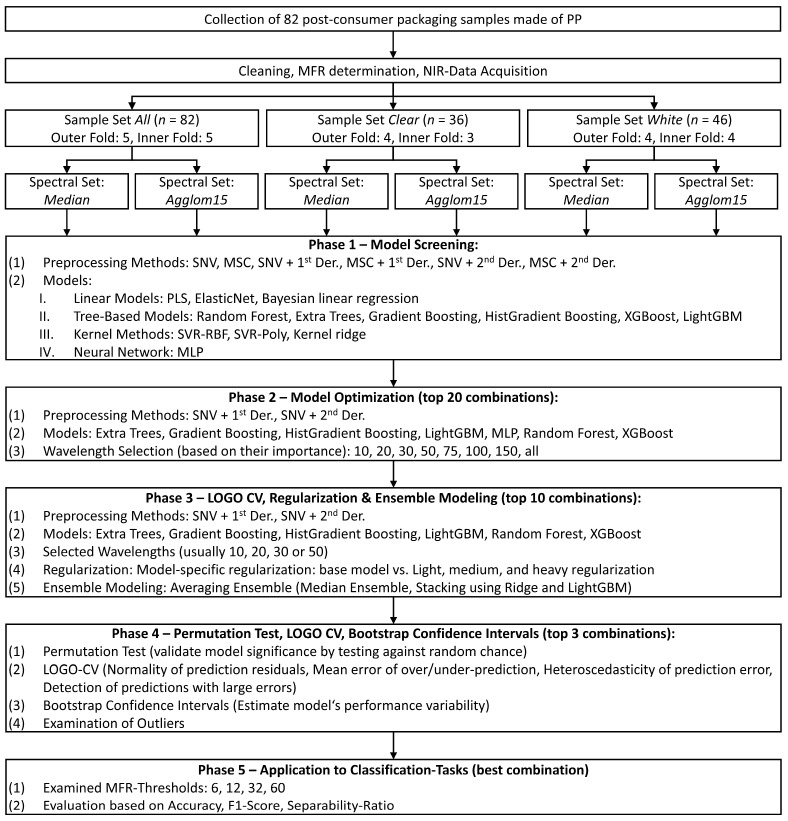
Process scheme summarizing the main steps from sample acquisition and MFR determination with an emphasis on modeling. Modeling comprised of model screening to find best performing combinations of model architectures as well as preprocessing, model optimization to combine best performing model combinations with feature (wavelength band) extraction, a regularization and ensemble modeling phase, to further decrease model generalization error and increase model performance, a robustness phase to validate the best performing model and examine their capabilities as well as a classification phase where the best performing models were used to classify at different MFR-thresholds.

Given the structural diversity within commercial PP (homopolymer, random copolymer, block copolymer), models were developed under two scenarios reflecting practical recycling constraints: The first scenario comprises all 82 samples (clear and white) and we refer to it as sample set *all*; this approach is closer to industrial practice as both are usually jointly processed. The second scenario comprises the samples separated by translucency (sample set *clear*, *n* = 36) versus opacity (sample set *white*, *n* = 46), motivated by evidence that optical pre-sorting improves the mechanical properties of PP recyclates [44]. Both scenarios were conducted through all modeling stages described below. An overview of the complete workflow is provided in Figure 2.

#### 2.2.1. Regression Modeling

To reduce computational load while preserving spectral variability, two spectral representations were derived for each sample. The spectral subset *median* comprises a single median spectrum, computed as the pixel-wise median across all retained spectra per sample. The spectral subset agglom15 includes fifteen representative spectra per sample, selected via agglomerative clustering to capture intra-sample heterogeneity.

For each scenario (sample set *all*, *clear*, *white*) as well as each spectral subset (*median*, *agglom15*), six preprocessing pipelines were evaluated applying preprocessing steps such as standard normal variate (SNV), multiplicative signal correction (MSC), and Savitzky–Golay derivatives:

- SNV
- SNV + 1st derivative (Savitzky–Golay, window size: 13; order: 3)
- SNV + 2nd derivative (Savitzky–Golay, window size: 15; order: 3)
- MSC
- MSC + 1st derivative (Savitzky–Golay, window size: 13; order: 3)
- MSC + 2nd derivative (Savitzky–Golay, window size: 15; order: 3)

While SNV reduces multiplicative spectral effects through row-wise scaling, MSC reduces baselines and multiplicative spectral variations through linear least-squares fitting of a “reference spectrum” to each spectrum [25,26]. Derivative filters such as Savitsky–Golay not only remove baseline offset and slope effects in spectra, but also improve spectral features and thus their discriminability [25,26]. For each pipeline, we additionally tested dimensionality reduction via principal component analysis (PCA), retaining the first 20 components. To address skewness and high variance in the target variable, models were also trained with and without a log-transformation of MFR.

A grid search evaluated 13 regression architectures spanning linear, tree-based, kernel, and neural-network families. Parameter settings are listed in *Appendix A*.

- *Linear Models*: Partial Least Squares (PLS) Regression, Elastic Net Regression, Bayesian Ridge Regression
- *Tree-Based Models*: Random Forest, Extremely Randomized Trees (ExtraTrees), Gradient Boosting Machines, Histogram-based Gradient Boosting, Extreme Gradient Boosting (XGBoost), Light Gradient Boosting Machine (LightGBM)
- *Kernel Methods*: Support Vector Regression—RBF kernel (SVR-RBF), Support Vector Regression—Polynomial kernel (SVR-Poly), Kernel Ridge Regression
- *Neural Network*: Multilayer Perceptron (MLP)

Combined with two spectral representations, six preprocessing pipelines, PCA (yes/no), and log-transform (yes/no), this yielded 2 × 6 × 2 × 2 × 13 = 624 unique combinations, each evaluated for *all* samples, *clear* samples only, and *white* samples only. Combinations were ranked by cross-validated root mean squared error (RMSECV), and the 20 top-performing combinations were advanced to optimization.

In the subsequent optimization phase, the 20 top-performing combinations were subject to hyperparameter optimization. Afterwards, feature-importance-based wavelengths were extracted for each model and subsets of the top 10, 20, 30, 50, 75, 100, and 150 bands were compared against using the full spectral range. The settings represented among the optimized combinations were:

- *Spectral Subset*: median, agglom15
- *Preprocessing*: SNV + 1st derivative, SNV + 2nd derivative
- *Log-Transform*: Yes, No
- *Architectures*: ExtraTrees, GradientBoosting, HistGradientBoosting, LightGBM, MLP, RandomForest, XGBoost

Afterwards, the 20 optimized combinations were evaluated for robustness via Leave-One-Group-Out (LOGO) cross-validation, grouping by sample identity in a refinement phase. Combinations were re-ranked by RMSECV, and the 10 best were subjected to regularization. Three regularization intensities were tested (“light,” “medium,” “heavy”), with architecture-specific parameters (e.g., α and λ for Elastic Net; tree depth and minimum samples per leaf for tree-based models; see Table A1 for full parameterization).

Lastly, the five best models were examined through permutation testing, where statistical significance was assessed via 100 permutations (α = 0.05). Next, the prediction stability was investigated through LOGO-CV to quantify variance in predictions. Afterwards, residuals were explored through Normality (Shapiro–Wilk), systematic bias (mean residual), and heteroscedasticity (residuals vs. predicted values). Outliers were detected from the prediction errors using a threshold of ±2 standard deviations of the errors. Lastly, out-of-bag bootstrapping (*n* = 1000) was used to estimate 95% confidence intervals on RMSECV and R^2^CV.

To investigate whether prediction outliers exhibited anomalous spectral characteristics, we compared their preprocessed spectra to those of samples with similar MFR (within ±15 g 10 min^−1^). For each outlier, z-scores were calculated per wavelength relative to the mean and standard deviation of the comparison group. Samples with low mean z-scores and few wavelengths exceeding 2 were classified as spectrally indistinguishable from their MFR-matched peers, indicating that prediction errors arose from model limitations rather than spectral anomalies.

#### 2.2.2. Classification Modeling

To assess model suitability for sensor-based sorting, the best-performing regression model was applied to binary classification. Because MFR is continuous, class boundaries must be defined a priori. Reflecting typical industrial sorting, which splits a bulk stream into two fractions, we evaluated binary classification at four MFR thresholds: 6, 12, 32, and 60 g 10 min^−1^. These thresholds were selected based on the observation that post-consumer PP recyclates typically exhibit MFR values between 13 and 20 g 10 min^−1^ [46], with optimized sorting achieving values near 12 g 10 min^−1^ [44] and dedicated hand sorting achieving values below 6 g 10 min^−1^ [47]. The threshold value of 32 g 10 min^−1^ was chosen because it lies between the mean and median of the sample set *all*, while the upper threshold of 60 g 10 min^−1^ corresponds to its 75th percentile. For each threshold, classification performance was quantified via accuracy (proportion of correctly classified samples) as well as the F1-score (harmonic mean of precision and recall).

### 2.3. Software

All analyses were performed in Python 3.13. Preprocessing and modeling relied on scikit-learn 1.8.0, XGBoost 3.1.3, and LightGBM 4.6.0. Visualization was performed with matplotlib 3.9.3 and PtitPrince 0.3.1. Hyperspectral data was extracted using HAIP BlackStudio 0.2.127. Generative artificial intelligence (Claude Opus, 4.5) has been used in this paper to assist during data analysis.

## 3. Results and Discussion

### 3.1. Sample Collection and Preparation

**Figure 3:**
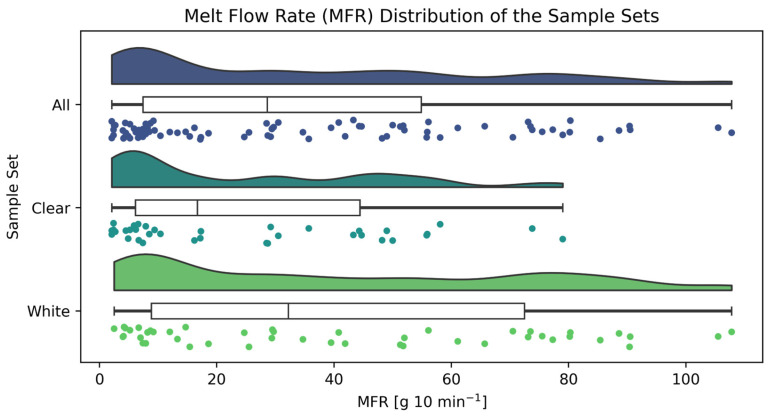
MFR distribution across the sample sets *all* (*n* = 82, min = 2.1 g 10 min^−1^, max = 107.8 g 10 min^−1^, mean = 34.0 g 10 min^−1^, median = 28.6 g 10 min^−1^), *clear* (*n* = 36, min = 2.1 g 10 min^−1^, max = 79.0 g 10 min^−1^, mean = 25.0 g 10 min^−1^, median = 16.7 g 10 min^−1^), and *white* (*n* = 46, min = 2.5 g 10 min^−1^, max = 107.8 g 10 min^−1^, mean = 41.0 g 10 min^−1^, median = 32.0 g 10 min^−1^).

The collected sample set comprised 82 rigid PP packages spanning a broad MFR range representative of commercial packaging grades (Figure 3). Across *all* samples, MFR ranged from 2.1 to 107.8 g 10 min^−1^ (mean 34.0 ± 29.5 g 10 min^−1^, median 28.6 g 10 min^−1^), with a moderate positive skew (0.71) indicating a higher frequency of lower-MFR samples.

*Clear* and *white* samples differed notably in their MFR distributions. *Clear* samples (*n* = 36) exhibited a narrower range (2.1–79.0 g 10 min^−1^) and lower central tendency (mean 25.0 g 10 min^−1^, median 16.7 g 10 min^−1^) compared to *white* samples (*n* = 46), which spanned 2.5–107.8 g 10 min^−1^ (mean 41.0 g 10 min^−1^, median 32.2 g 10 min^−1^). Both subsets displayed positive skewness (*clear*: 0.74; *white*: 0.43), though the effect was more pronounced in the *clear* subset. These distributional differences support the decision to evaluate combined and separated modeling scenarios, as the spectral-MFR relationship may differ between opacity classes.

The observed MFR range aligns with values reported for post-consumer PP recyclates in the literature, which typically center around 10–20 g 10 min^−1^ but can extend considerably higher depending on feedstock composition [44,46,47].

### 3.2. Experimental Design and Sample Grouping

This section includes the results from regression and classification modeling.

#### 3.2.1. Regression Modeling

The screening phase evaluated 624 unique combinations of preprocessing, dimensionality reduction, target transformation, spectral representation, and model architecture across three sample sets. The 20 top-performing combinations per sample set are reported in Table A2. Several consistent patterns emerged:

Across the sample set *all* (*n* = 82), the best-performing combination achieved RMSECV = 19.2 g 10 min^−1^ and R^2^CV = 0.57 (ExtraTrees, SNV + 1st derivative, *median* spectrum). Tree-based ensemble methods dominated the top 20, with ExtraTrees, gradient boosting variants (HistGradientBoosting, LightGBM, XGBoost), and Random Forest appearing consistently. First-derivative preprocessing outperformed second-derivative preprocessing at this stage, and both SNV and MSC performed comparably. Neither PCA-based dimensionality reduction nor log-transformation of MFR improved performance for this sample set.

For the sample set *clear* (n = 36), the modeling results are notably lower: the best combination achieved RMSECV = 17.6 g 10 min^−1^ but R^2^CV = 0.36 (ExtraTrees, MSC + 1st derivative, agglom15). The lower R^2^ despite similar absolute RMSE reflects the narrower MFR variance in the *clear* subset (SD = 22.4 vs. 29.5 g 10 min^−1^ overall). Interestingly, all 20 top-performing combinations for *clear* samples used the agglom15 spectral representation rather than *median* spectra, suggesting that capturing intra-sample spectral heterogeneity is beneficial when samples are optically more homogeneous. Log-transformation appeared only once in the top 20, indicating limited benefit.

For *white* samples (n = 46), the model screening yielded substantially better performance: the best combination achieved an RMSECV of 15.3 g 10 min^−1^ and an R^2^CV of 0.76 (LightGBM, MSC + 2nd derivative, *median* spectrum, log-transformed MFR). In contrast to *clear* samples, all top 20 combinations for *white* samples used median spectra, and log-transformation appeared frequently (9 of 20 combinations), suggesting that the higher MFR variance and positive skew in this subset benefited from variance-stabilizing transformation. Second-derivative preprocessing appeared more frequently than for other subsets.

These screening results indicate that there is a spectral-MFR relationship (RQ1), which is stronger in *white* PP than in *clear* PP. However, the RMSE is considerably high across all sample sets. Furthermore, the optimal spectral representation in terms of median spectrum versus single spectra also differs between *white* PP and *clear* PP. This suggests greater spectral variability within the *white* PP, which may make model fitting more difficult as information may be unevenly distributed within the spectral pixels. Because the two spectral representation strategies have different practical implications (assessing a flake according to its median spectrum versus by its individual spectral pixels), both approaches were investigated further. Although MSC and SNV often give comparable corrections for scattering, SNV was preferred in subsequent modeling because it operates on individual spectra. MSC requires a reference spectrum from the calibration set, whereas SNV can be applied per sample, which simplifies transferring the model to online settings where single-sample predictions are needed [26]. PCA-based post-processing did not appear among the best results, so PCA was omitted in the next steps. The log-transformation of the target variable showed a positive effect only for the *white* sample set, even though the target skewness is similar across the three sets.

Interestingly, tree-based architectures consistently outperform linear models as well as neural networks. This implies the spectrum-MFR relationship is not purely linear. Tree models may perform well because they can represent high-dimensional but sparse/informative features as many simple rules and thus handle signals buried in noise [48]. Moreover, this contrasts the findings of [45] who found a strong linear relationship using PLS.

**Figure 4:**
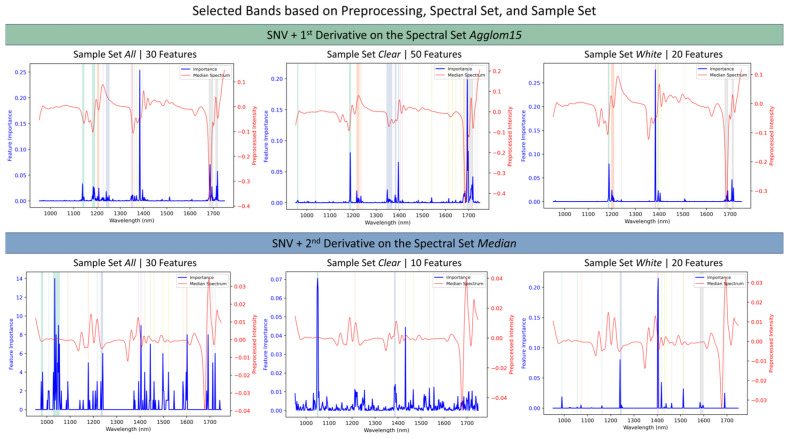
Computed median spectrum per sample set (red) and selected bands (vertical bars) together with the relative importance of such bands (blue) during the optimization phase. Each plot illustrates the optimal combination of band selection and preprocessing methods (SNV + 1st derivative (**top row**), SNV + 2nd derivative (**bottom row**)), respectively. The spectral set is represented by agglom15 (**top row**) and median (**bottom row**), while the sample set is shown as *all* (**left**), *clear* (**middle**), and *white* (**right**).

In the subsequent optimization phase, a wavelength selection based on feature importance was applied to the top 20 screening combinations. By reducing the input data to these selected spectral ranges, performance was generally improved or maintained, while the complexity was significantly reduced (Figure 4). Feature importance metrics were extracted from the respective models using their built-in importance scores computed during training.

Although increasing derivative order generates more localized band structures and thus may facilitate a better analysis, noise increases too. Within the top 20 screening combinations, no clear difference regarding the model performance arises according to the chosen preprocessing method. However, the feature importance scatters stronger when the second derivative is used, indicating that potentially relevant information might be blurred by instrument noise [26].

The fact that the most important information linking spectrum and MFR is concentrated in a few bands is evident from the fact that selected feature combinations contain only 10–30 bands. Interestingly, this band reduction benefits the sample set *clear* much stronger (ΔR^2^ ≈ +0.17) than the sample set *white* (ΔR^2^ ≈ +0.07). RMSE also decreases across *all* sample sets by roughly 0.03.

Comparing the chosen bands across sample sets, all models consistently select the characteristic polypropylene absorption bands (RQ2): Asymmetric methyl and methylene stretching (2nd overtone) between 1180 and 1250 nm, methyl and methylene combination around 1380–1420 nm, and asymmetric methyl and methylene stretching (1st overtone) between 1680 and 1750 nm [49]. This indicates the models are not merely fitting noise or spurious correlations; instead, the selected bands offer insight into the samples’ morphological and chemical/molecular structure and therefore help explain the link between vibrational information and recycling-relevant processing information. For example, ref. [32] state that peaks at 1691 and 1724 nm can be associated with the short helices linked to the amorphous domain of PP, while peaks observed at 1706 and 1736 nm can be assigned to long helices in the crystalline domain. Generally speaking, the increased γ-phase—stemming from the random incorporation of comonomers such as Ethylene in PP-random—translates not only mechanically to reduced stiffness but also perceptually to improved clarity [39]. Hence, one would expect that the models trained on *clear* PP exhibit increased sensitivity to the 1706 and 1736 nm bands while the models trained on *white* PP exhibit increased sensitivity to bands at 1691 and 1724 nm. And indeed, 50% of the models trained on *clear* selected the band 1706 nm, but only 10% selected the band at 1736 nm. On the other hand, 50% of the models trained on *white* PP selected the band at 1690 nm but none selected the band at 1724 nm, which in turn was selected by 35% of the *clear*-trained models. This could indicate the limitations of this rule of thumb and requires further characterization of the samples, e.g., through differential scanning calorimetry (DSC). However, it should be noted that the existing literature on the assignment of NIR bands to MFR-relevant factors is limited. This makes it difficult to conduct a more in-depth analysis. Examples of these factors include molar mass [42], molar mass distribution [42], and branching [43].

In the subsequent refinement phase, cross-validation was changed from the previous class-wise stratified *k*-fold iterator variant with non-overlapping groups (StratifiedGroupKFold) to a leave-one-group-out cross-validation (LOGO-CV) per sample set, with the re-ranking based on LOGO R^2^. This step revealed notable rank changes: for instance, in samples set *all*, the configuration ranked 7th in optimization (ExtraTrees, SNV + 2nd derivative, median) rose to rank 1 in LOGO-CV with only a 3.3% decrease in R^2^, while the former rank-1 configuration dropped to rank 2 with a 4.4% increase in apparent performance—indicating slight overfitting in the latter. Similar patterns were observed across sample sets, with XGBoost and LightGBM configurations tending to show larger generalization gaps than ExtraTrees and GradientBoosting.

Regularization was applied to the 10 best LOGO-CV configurations, testing light, medium, and heavy parameterizations. For most configurations, regularization did not substantially improve LOGO performance, suggesting that the tree-based ensembles with default hyperparameters were not severely overfitting. Ensemble strategies (median ensemble and stacking with Ridge or LightGBM meta-learners) were evaluated; for *white* samples, a median ensemble of GradientBoosting, HistGradientBoosting, and LightGBM achieved the best final performance. This suggests that the architectures identified during the screening phase were already appropriate for capturing the spectrum-MFR relationships, and that wavelength selection was the decisive step for extracting the relevant correlations. Regularization did not further reduce generalization error, which also points to model robustness; consequently, no further regression tuning was pursued.

The three best-performing models per sample set and spectral subset are listed in Table 1. It reveals pronounced differences in predictive performance between sample types and spectral representation (RQ3). For the combined sample set, the final model explains 66% of MFR variance with a prediction error of ±17.3 g 10 min^−1^. For *clear* samples, despite lower absolute RMSE (14.0 g 10 min^−1^), R^2^ is moderate (0.61), reflecting the lower MFR variance in this subset. For *white* samples, the ensemble model achieves RMSE = 12.4 g 10 min^−1^ with R^2^ = 0.85, representing substantially better predictive performance. This difference persists even when accounting for the narrower MFR variance and skew in the *clear* subset, suggesting a genuinely weaker spectral–MFR relationship rather than merely a statistical artifact. Several factors can contribute to this discrepancy. Opaque samples exhibit more uniform diffuse reflection, while transparent samples exhibit variable transmission and internal scattering depending on their thickness and surface properties. In fact, the individual spectral pixels in *clear* samples—especially in thin-walled thermoformed products—have more noise than in *white* samples, which can mask subtle artifacts [50]. On the other hand, however, a more homogeneous composition would be expected in terms of the PP types used, as these tend to comprise random copolymers; whereas, *white* can contain homopolymers, random copolymers and block copolymers. Finally, different additives could cause spectral properties that either reinforce or distort the MFR relationship. The weak performance on the *clear* sample set contrasts the findings of [45] who examined nine clear PP packaging items and reported strong correlations between NIR spectra and dynamic viscosity (R^2^ = 0.97) using PLS regression. This difference is rooted in the larger and more heterogeneous sample set (36 vs. 9 items), broader MFR range (2.1–79.0 vs. ~930–7300 Pa·s, assuming typical viscosity–MFR relationships), and inclusion of in-use post-consumer packaging rather than off-the-shelf products. These differences highlight the methodological challenges of generalizing findings to realistic recycling streams and, at the same time, the need for further in-depth investigation. Looking at studies on chemically similar polyethylene, ref. [33] reported successful NIR prediction of density, and [37] demonstrated prediction of crystallinity and short-chain branching. The performance achieved here for *white* PP (R^2^ = 0.85) is comparable to these PE studies, suggesting that NIR-based property prediction for PP is similarly feasible when optical interference is controlled.

The consistent superiority of median-spectrum representations over agglom15 in the final models—despite agglom15 performing well for *clear* samples during screening phase—suggests that pixel-level heterogeneity, while informative during initial training, introduces additional noise that impairs generalization in LOGO-CV. The median spectrum, by averaging intra-sample variability, may provide a more robust representation of each sample’s bulk chemical properties. For industrial implementation, this would necessitate a combination of object detection with prediction on median rather than the currently more often applied pixel-wise prediction.

**Figure 5:**
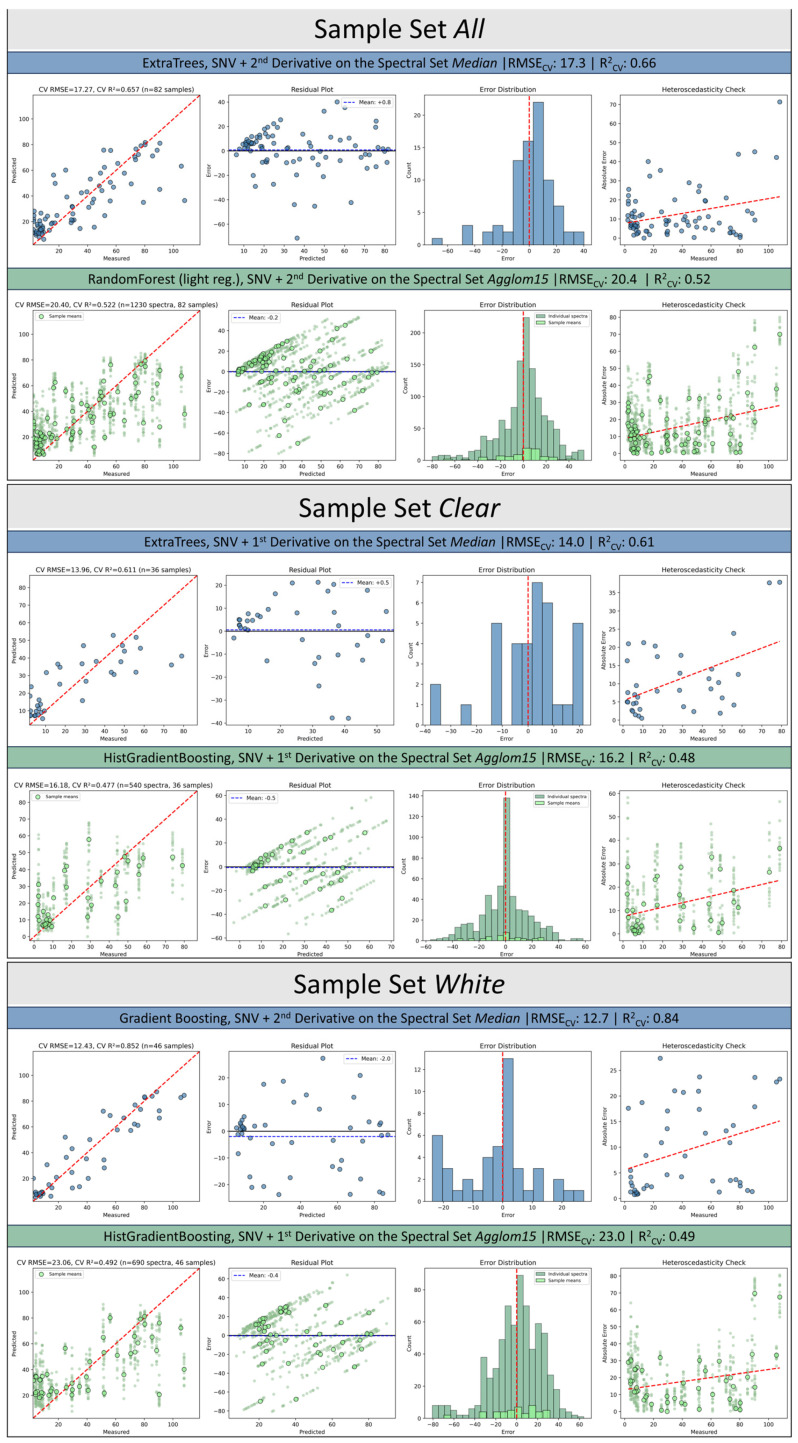
Results of the robustness phase depicted through a regression plot, residual plot, error distribution plot and a hydroelasticity plot for the three examined sample sets as well as for the respective best model for the spectral subset median and agglom15.

All final models passed permutation testing (*p* < 0.01), confirming that spectral-MFR relationships are statistically significant. However, residual diagnostics revealed consistent patterns across sample sets that inform model applicability (Figure 5).

For the combined sample set, the median-based ExtraTrees model achieved RMSE = 17.3 g 10 min^−1^ and R^2^ = 0.66 (bootstrap 95% CI: 0.34–0.80). Predicted-versus-measured plots showed a clear monotonic trend with most points near the 1:1 line, though the highest MFR values were systematically under-predicted, producing residuals up to −71 g 10 min^−1^. Residuals were unbiased (mean = +0.8) but exhibited heteroscedasticity (r = 0.31), with error magnitude increasing at higher MFR values. Six outliers were detected. The agglom15 representation performed notably worse (RMSE = 20.4, R^2^ = 0.52, bootstrap CI: 0.15–0.68), with stronger heteroscedasticity (r = 0.38) and greater compression of extreme values toward the mean—indicating that treating pixel-level replicates as independent observations introduces noise rather than improving signal. This means that chemically relevant information could also be masked by the high signal-to-noise ratio of a single spectral pixel and only become visible when the aggregated data is analyzed.

For *clear* samples, the median model achieved RMSE = 14.0 g 10 min^−1^ and R^2^ = 0.61, but with substantial instability (bootstrap CI: −0.26 to 0.77) reflecting the small sample size (n = 36). Heteroscedasticity was most pronounced in this subset (r = 0.50): low-MFR samples were predicted with reasonable accuracy, while high-MFR samples exhibited large errors. Two persistent outliers dominated the error distribution. The agglom15 model showed further degradation (R^2^ = 0.48, bootstrap CI: −0.70 to 0.68), confirming that median aggregation is preferable for this optically challenging subset.

For *white* samples, the median-based ensemble achieved the strongest and most stable performance (RMSE = 12.7, R^2^ = 0.84, bootstrap CI: 0.32–0.83). Predicted values aligned tightly with measurements across the full MFR range, with only one prominent outlier and moderate heteroscedasticity (r = 0.34). In contrast, the agglom15 representation degraded markedly (RMSE = 23.0, R^2^ = 0.49), with pronounced shrinkage toward the mean and substantially wider bootstrap intervals.

Across all sample sets, common patterns emerged: no systematic bias, non-normal but symmetric residual distributions, and heteroscedasticity indicating reduced reliability at high MFR values. Median spectral representations consistently outperformed agglom15, and the same problematic samples appeared as outliers across models, suggesting an irreducible error floor inherent to the spectral approach

Outlier analysis revealed two distinct categories of poorly predicted samples. Some outliers showed significant spectral deviations from MFR-matched samples, suggesting compositional differences or unusual additive formulations. However, other outliers were spectrally indistinguishable from samples with similar MFR (±10 g 10 min^−1^), yet exhibited large prediction errors—demonstrating that MFR differences can exist without corresponding spectral signatures detectable by NIR-HSI, and highlighting an inherent limitation of the spectroscopic approach.

#### 3.2.2. Classification Modeling

Due to the high RMSE in all sample sets and the resulting limited suitability for use in quality control (e.g., for the assessment of incoming batches), the suitability of the best regression models for binary classification tasks as used in sensor-based sorting was investigated, too. For this, threshold values were defined at 6, 12, 32, and 60 g 10 min^−1^, spanning the practical range for PP packaging recyclates, with 12 g 10 min^−1^ representing an achievable target for high-quality recyclates and 32 g 10 min^−1^ approximating the median MFR of the combined sample set. Classification performance was evaluated using accuracy, balanced accuracy, and macro-averaged F1-score; results are summarized in Table 2.

Classification performance varied substantially with threshold selection, reflecting both the underlying MFR distribution and the regression model’s predictive accuracy across the MFR range (RQ4): For the combined sample set (*n* = 82), the threshold at 32 g 10 min^−1^ yielded optimal performance (balanced accuracy = 0.90, F1 = 0.90), with errors evenly distributed between false positives and false negatives. This threshold lies close to the sample median (28.6 g 10 min^−1^), resulting in relatively balanced class sizes and symmetric error distribution. At lower thresholds (6 and 12 g 10 min^−1^), performance degraded substantially: balanced accuracy dropped to 0.50–0.70, driven by high false-positive rates. At these thresholds, the minority class (samples with MFR < threshold) contains few members, and the model systematically over-predicts MFR for these samples. The threshold at 60 g 10 min^−1^ maintained reasonable performance (balanced accuracy = 0.82), though false negatives began to increase as high-MFR samples were under-predicted which is in turn consistent with the heteroscedasticity observed in regression residuals.

For *clear* samples (*n* = 36), the threshold at 12 g 10 min^−1^ yielded best performance (balanced accuracy = 0.82, F1 = 0.82), reflecting the lower median MFR of this subset (16.7 g 10 min^−1^). At the 32 g 10 min^−1^ threshold, performance remained acceptable (balanced accuracy = 0.79). However, extreme thresholds performed poorly: at 6 g 10 min^−1^, balanced accuracy was only 0.48, and at 60 g 10 min^−1^, it dropped to 0.50 despite high overall accuracy (0.94)—a misleading metric in this case, as only two samples in the *clear* subset exceeded 60 g/10 min, and both were misclassified. Again, this highlights the importance of selecting thresholds appropriate to the MFR distribution of the target population.

**Figure 6:**
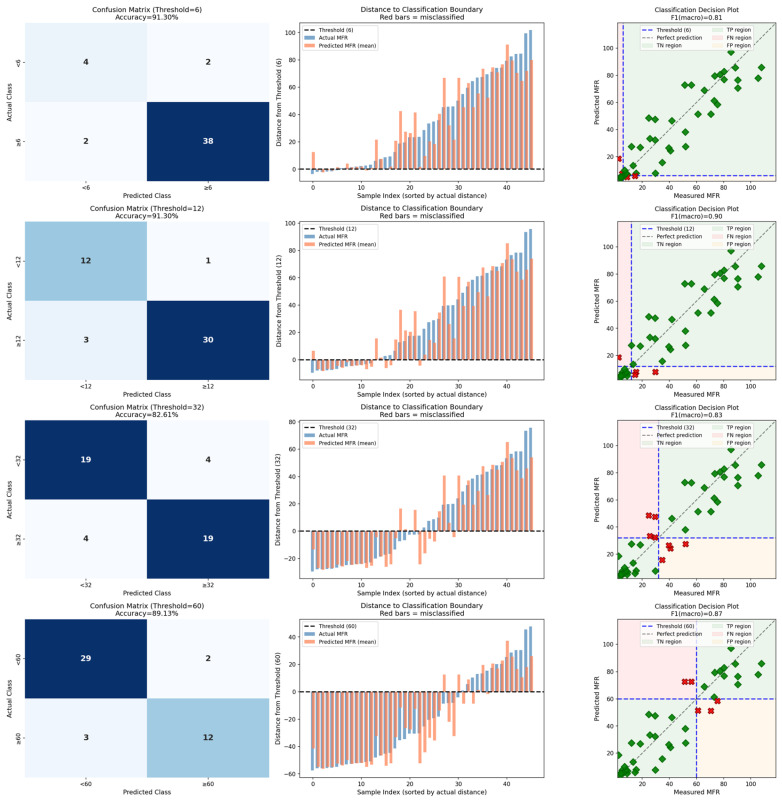
Results of the classification phase depicted through a confusion matrix, distance to classification boundary, and classification decision plot for the best performing model (sample set *white*, spectral subset median) at the thresholds 6, 12, 30, and 60 g 10 min^−1^. In the classification decision plot, green diamonds indicate correctly classified samples, while red crosses indicate misclassified samples.

For *white* samples (*n* = 46), classification performance was consistently strong across all thresholds (Figure 6). The best performance was achieved at 12 g 10 min^−1^ (balanced accuracy = 0.92, F1 = 0.90), but performance remained high at all other thresholds (balanced accuracy 0.81–0.87). This robustness reflects the superior regression performance for *white* samples (LOGO R^2^ = 0.85) and suggests that NIR-based sorting of opaque PP by MFR is practically feasible across a range of industrially relevant thresholds.

Consistent with regression findings, median spectral representations outperformed agglom15 for classification across all sample sets and thresholds. For the combined sample set at the 32 g 10 min^−1^ threshold, median-based classification achieved F1 = 0.90 compared to F1 = 0.83 for agglom15. The performance gap was particularly pronounced for *white* samples: at the 12 g 10 min^−1^ threshold, median-based classification achieved balanced accuracy of 0.92, while agglom15 achieved only 0.54. This disparity likely reflects the increased noise introduced by pixel-level spectral variability when applied to classification, where decision boundaries are more sensitive to prediction variance than regression metrics.

Although the total sample size of 36 *clear* and 46 *white* and sample was bigger than in most comparable studies (e.g., [37,45]), the bootstrap confidence intervals still indicate substantial uncertainty in performance estimates (95% CI width 59–86% for R^2^). Hence, larger sample sets would provide more reliable performance estimates and enable investigation of subgroup effects (e.g., by PP type or application category). Moreover, validating on a larger, independent dataset would further strengthen confidence in the generalizability of the finding and enable more reliable uncertainty quantification. Although colored packaging was intentionally excluded to avoid confounding by additives, colored PP constitutes a substantial fraction of the recyclate stream and warrants separate investigation.

Moreover, it is quite conceivable that the ambiguity of the results can also be linked to the choice of MFR as the target parameter. Although this is relevant to industry, it is an indirect indicator of processability that integrates multiple chemical and physical factors such as molar mass [42], molar mass distribution [42], branching [43], and fillers [51]. It is conceivable that alternative target parameters—such as direct molar mass or melt viscosity at processing-relevant shear rates—provide spectrally more interpretable relationships. This is evident, for example, in the outliers present in each sample set: despite similar MFRs, these showed significant spectral differences or differed in their spectral characteristics but not in their MFR.

Finally, examining complementary spectral ranges—particularly in the upper part of the shortwave infrared range (SWIR, 1750–2500 nm) or the mid-infrared range (MIR, 2500–5000 nm)—could reveal additional informative features or improve prediction accuracy. Another aspect would be the reduction in the spectral range in favor of increased spectral resolution. This could enable the targeted investigation of specific, promising band ranges with potential fine spectral artifacts (e.g., peak ratios).

## 4. Conclusions

This study systematically evaluated NIR-HSI for predicting melt flow rate in post-consumer PP packaging. A statistically significant relationship exists between NIR spectra and MFR (all models *p* < 0.01), though its strength varies by sample opacity/translucency (RQ1, RQ2). *Clear* samples reached only R^2^ = 0.61 (RMSE = 14.0 g 10 min^−1^) while *white* samples achieved R^2^ = 0.85 (RMSE = 12.4 g 10 min^−1^), and combined samples R^2^ = 0.66 (RMSE = 17.3 g 10 min^−1^). The weaker relationship in *clear* samples likely reflects different optical properties affecting spectral consistency and/or interfering effects from additives.

Feature-importance analysis identified key regions associated with MFR prediction that consistently match the characteristic polypropylene absorption bands (RQ2): Asymmetric methyl and methylene stretching (2nd overtone) between 1180 and 1250 nm, methyl and methylene combination around 1380–1420 nm, and asymmetric methyl and methylene stretching (1st overtone) between 1680 and 1750 nm. In general, it is difficult to establish a direct link between specific NIR bands and factors influencing MFR due to a lack of literature.

Besides sample opacity/translucency, median spectral representations outperformed pixel-level (agglom15) aggregation across all sample sets and modeling phases, indicating that averaging intra-sample variability provides more robust predictions than preserving pixel-level heterogeneity (RQ3). This implies that decision boundaries are more sensitive to prediction variance than regression metrics.

In conclusion, we found that the industrial applicability of NIR spectra for predicting MFR in regression tasks was lower than in classification tasks, due to the high RMSE value of the model (RQ4). However, with balanced accuracies of 0.82 to 0.92, classification appears applicable even at low MFR limits (6 and 12 g 10 min^−1^). This suggests that it is a viable option for industrial practice, with the potential to enhance the substitutability of recycled polypropylene.

## Figures and Tables

**Table 1:** Summary of the performances after regression modeling by sample set, preprocessing, and spectral subset in terms of LOGO RMSE and LOGO R^2^. Best results were achieved when training on the spectral subset median and the sample set *white*.

Sample Set	Preprocessing	Spectral Subset	Regularization/Ensemble	Model	LOGO RMSE	LOGO R^2^
*All*	SNV + 2nd Der	median	None	ExtraTrees	17.3	0.66
*All*	SNV + 1st Der	median	None	ExtraTrees	17.4	0.65
*All*	SNV + 2nd Der	median	Ensemble	Stacking Ridge	17.4	0.65
*All*	SNV + 2nd Der	agglom15	Light	RandomForest	20.4	0.52
*All*	SNV + 1st Der	agglom15	Light	RandomForest	20.4	0.52
*All*	SNV + 2nd Der	agglom15	Ensemble	RandomForest	20.4	0.52
*Clear*	SNV + 1st Der	median	None	ExtraTrees	14.0	0.61
*Clear*	SNV + 1st Der	median	Ensemble	ExtraTrees	14.0	0.61
*Clear*	SNV + 2nd Der	median	None	ExtraTrees	14.1	0.60
*Clear*	SNV + 2nd Der	agglom15	None	LightGBM	16.2	0.48
*Clear*	SNV + 1st Der	agglom15	None	HistGradientBoosting	16.2	0.48
*Clear*	SNV + 1st Der	agglom15	None	HistGradientBoosting	16.2	0.48
*White*	SNV + 2nd Der	median	Ensemble	GradientBoosting, HistGradient-Boosting, Light-GBM	12.4	0.85
*White*	SNV + 2nd Der	median	Ensemble	GradientBoosting, HistGradient-Boosting, Light-GBM	12.4	0.85
*White*	SNV + 2nd Der	median	None	GradientBoosting	12.7	0.84
*White*	SNV + 1st Der	agglom15	None	HistGradientBoosting	23.0	0.49
*White*	SNV + 1st Der	agglom15	None	RandomForest	23.1	0.49
*White*	SNV + 1st Der	agglom15	None	XGBoost	23.2	0.49

**Table 2:** Results of the classification modeling using the best regression model for each per sample set and spectral subset. Each model was applied at different MFR-thresholds (6, 12, 32, 60 g 10 min^−1^) and evaluated according to its accuracy, balanced accuracy, F1 macro (weighted) and share of misclassified samples.

Sample Set	Spectral Subset	Preprocessing	Model Name	MFR-Threshold	Accuracy	Balanced Accuracy	F1 Macro	Misclassified Samples [%]
*All*	median	SNV + 2nd Der	ExtraTrees	6	0.82	0.50	0.73	18
				12	0.78	0.70	0.75	22
				32	0.90	0.90	0.90	10
				60	0.89	0.82	0.89	11
	agglom15	SNV + 2nd Der	RandomForest	6	0.82	0.50	0.73	18
				12	0.73	0.63	0.68	27
				32	0.83	0.83	0.83	17
				60	0.85	0.71	0.84	15
*Clear*	median	SNV + 1st Der	ExtraTrees	6	0.72	0.48	0.63	28
				12	0.83	0.82	0.83	17
				32	0.81	0.79	0.81	19
				60	0.94	0.50	0.92	6
	agglom15	SNV + 1st Der	HistGradientBoosting	6	0.75	0.50	0.64	25
				12	0.81	0.80	0.80	19
				32	0.78	0.75	0.78	22
				60	0.94	0.50	0.92	6
*White*	median	SNV + 2nd Der	GradientBoosting	6	0.91	0.81	0.91	9
				12	0.91	0.92	0.91	9
				32	0.83	0.83	0.83	17
				60	0.89	0.87	0.89	11
	agglom15	SNV + 1st Der	HistGradientBoosting	6	0.87	0.50	0.81	13
				12	0.74	0.54	0.65	26
				32	0.80	0.80	0.80	20
				60	0.78	0.70	0.77	22

## Data Availability

The data presented in this study are available on request from the corresponding author.

## Abbreviations

The following abbreviations are used in this manuscript:

BM	Blow Molding
DSC	Differential scanning calorimetry
ExtraTrees	Extremely Randomized Trees
HSI	Hyperspectral imaging
IM	Injection Molding
LightGBM	Light Gradient Boosting Machine
LOGO	Leave-One-Group-Out
MFR	Melt Flow Rate
MIR	Mid-infrared
MLP	Multilayer Perceptron
MRF	Material Recovery Facility
MSC	Multiplicative signal correction
NIR	Near-infrared
PCA	Principal Component Analysis
PE	Polyethylene
PET	Polyethylene terephthalate
PLS	Partial Least Square
PP	Polypropylene
PPWR	Packaging and Packaging Waste Regulation
RMSE	Root mean squared error
ROI	Regions of interest
SNV	Standard normal variate
SVR-Poly	Support Vector Regression-Polynomial kernel
SVR-RBF	Support Vector Regression-RBF kernel
SWIR	Shortwave infrared range
TF	Thermoforming
XGBoost	Extreme Gradient Boosting

## Appendix A

### Parameter Settings During Grid Search

The parameter settings listed below were used for the evaluated 13 regression architectures during the grid search:

*Linear Models*:

- Partial Least Squares (PLS) Regression: *n* of components: 2, 3, 5, 7, 10, 15, 20
- Elastic Net Regression: Max. iterations: 5000; alpha: 0.01, 0.1, 1.0; l1-Ratio: 0.2, 0.5, 0.8
- Bayesian Ridge Regression: alpha_1: 1 × 10^−6^, 1 × 10^−5^; lambda_1: 1 × 10^−6^, 1 × 10^−5^

*Tree-Based Models*:

- RandomForest: Number of estimators: 200, 500; min. samples leaf: 1, 3, 5; max. features: square root, 0.3
- Extremely Randomized Trees: Number of estimators: 200, 500; min. samples leaf: 1, 3, 5; max. features: square root, 0.3
- Gradient Boosting Machines: Number of estimators: 200; learning rate: 0.05, 0.1; max. depth: 3, 5; min. samples leaf: 3, 5
- Histogram-based Gradient Boosting: Max. iterations: 200; learning rate: 0.05, 0.1; max. depth: 5, 10; min. samples leaf: 5, 10
- Extreme Gradient Boosting: Number of estimators: 200, 500; learning rate: 0.03, 0.05, 0.1; max. depth: 3, 5, 7; min. child weight: 1, 3, 5; subsample: 0.8; colsample by tree: 0.8
- Light Gradient Boosting Machine: Number of estimators: 200, 500; learning rate: 0.03, 0.05, 0.1; max. depth: 3, 5, 7; min. child samples: 5, 10; subsample: 0.8; colsample by tree: 0.8

*Kernel Methods*:

- Support Vector Regression—RBF kernel (SVR-RBF): Coefficient: 1, 10, 100, 1000; Gamma: “scale”, 1 × 10^−3^, 1 × 10^−2^
- Support Vector Regression—Polynomial kernel (SVR-Poly): Coefficient: 1, 10, 100; degree: 2, 3; gamma: “scale”
- Kernel Ridge Regression: alpha: 1 × 10^−4^, 1 × 10^−3^, 1 × 10^−2^, 0.1; gamma: 1 × 10^−3^, 1 × 10^−2^, 0.1

*Neural Network*:

- Multilayer Perceptron: Hidden layer sizes: 100, 200, (100, 50); alpha: 1 × 10^−4^, 1 × 10^−3^, 1 × 10^−2^

**Table A1:** Regularization parameters used during the regularization phase.

Model	Estimators	Learning Rate	Max. Depth	Minimum -Child Samples -Child Weight -Samples Leaf	Min. Sample Split	Sub Sample	Sample by Tree/Max. Features	Reg. Alpha	Reg. Lambda
LightGBM: base	200	0.05	5	10	N/A	0.8	0.8	0	0
LightGBM: light_reg	300	0.03	4	20	N/A	0.7	0.7	0.1	0.1
LightGBM: medium_reg	500	0.01	3	30	N/A	0.6	0.6	1	1
LightGBM: heavy_reg	800	0.005	2	50	N/A	0.5	0.5	5	5
XGBoost: base	200	0.05	5	3	N/A	0.8	0.8	0	1
XGBoost: light_reg	300	0.03	4	5	N/A	0.7	0.7	0.1	2
XGBoost: medium_reg	500	0.01	3	10	N/A	0.6	0.6	1	5
XGBoost: heavy_reg	800	0.005	2	20	N/A	0.5	0.5	5	10
ExtraTrees: base	200	N/A	None	1	2	N/A	“sqrt”	N/A	N/A
ExtraTrees: light_reg	300	N/A	20	3	5	N/A	0.5	N/A	N/A
ExtraTrees: medium_reg	500	N/A	10	5	10	N/A	0.3	N/A	N/A
ExtraTrees: heavy_reg	500	N/A	5	10	20	N/A	0.2	N/A	N/A
RandomForest: base	200	N/A	None	1	2	N/A	“sqrt”	N/A	N/A
RandomForest: light_reg	300	N/A	20	3	5	N/A	0.5	N/A	N/A
RandomForest: medium_reg	500	N/A	10	5	10	N/A	0.3	N/A	N/A
RandomForest: heavy_reg	500	N/A	5	10	20	N/A	0.2	N/A	N/A

N/A = Not applicable. “sqrt” = Number of features used when looking for the best split; applied on n_features.

**Table A2:** Top 20 results of the screening phase according to all samples as well as separated by translucency (clear) and opacity (white).

Sample Set	Rank	Preprocessing	Post Processing	Spectral Subset	Transform	Model Name	RMSE (Mean)	RMSE (STD)	R^2^ (Mean)	R^2^ (STD)
all	1	SNV + 1st Der	None	median	none	ExtraTrees	19.2	2.4	0.57	0.09
all	2	MSC + 1st Der	None	median	none	ExtraTrees	19.4	2.4	0.56	0.11
all	3	MSC + 2nd Der	None	agglom15	none	HistGradientBoosting	19.6	3.3	0.55	0.12
all	4	MSC + 2nd Der	None	agglom15	none	LightGBM	19.6	3.5	0.55	0.11
all	5	MSC + 1st Der	None	agglom15	none	HistGradientBoosting	19.8	1.8	0.54	0.09
all	6	MSC + 1st Der	None	median	none	XGBoost	19.9	3.2	0.53	0.16
all	7	SNV + 1st Der	None	agglom15	none	XGBoost	19.9	2.2	0.53	0.13
all	8	SNV + 1st Der	None	median	none	LightGBM	20.0	4.0	0.50	0.27
all	9	SNV + 1st Der	None	agglom15	none	RandomForest	20.1	2.4	0.52	0.12
all	10	MSC + 1st Der	None	agglom15	none	XGBoost	20.1	1.9	0.52	0.15
all	11	SNV + 1st Der	None	agglom15	none	GradientBoosting	20.2	3.1	0.51	0.15
all	12	MSC + 1st Der	None	agglom15	none	LightGBM	20.2	2.5	0.51	0.15
all	13	MSC + 2nd Der	None	agglom15	none	XGBoost	20.2	2.8	0.52	0.11
all	14	SNV + 1st Der	None	agglom15	none	HistGradientBoosting	20.2	2.4	0.51	0.13
all	15	MSC + 1st Der	None	agglom15	none	ExtraTrees	20.2	2.4	0.51	0.12
all	16	SNV + 1st Der	None	agglom15	none	LightGBM	20.2	2.5	0.51	0.16
all	17	MSC + 1st Der	None	agglom15	none	RandomForest	20.4	2.7	0.51	0.13
all	18	MSC + 1st Der	None	median	none	LightGBM	20.4	3.3	0.50	0.19
all	19	MSC + 2nd Der	None	agglom15	none	GradientBoosting	20.5	3.3	0.51	0.12
all	20	SNV + 1st Der	None	agglom15	none	ExtraTrees	20.5	2.7	0.50	0.15
clear	1	MSC + 1st Der	None	agglom15	none	ExtraTrees	17.6	3.2	0.36	0.17
clear	2	SNV + 1st Der	None	agglom15	none	GradientBoosting	17.7	4.5	0.36	0.21
clear	3	MSC + 2nd Der	None	agglom15	none	ExtraTrees	17.8	3.4	0.34	0.18
clear	4	MSC + 1st Der	None	agglom15	none	HistGradientBoosting	17.9	3.7	0.34	0.20
clear	5	SNV + 1st Der	None	agglom15	none	RandomForest	17.9	3.2	0.34	0.16
clear	6	SNV + 1st Der	None	agglom15	none	XGBoost	18.0	3.8	0.34	0.17
clear	7	MSC + 1st Der	None	agglom15	none	GradientBoosting	18.1	2.8	0.32	0.17
clear	8	MSC + 2nd Der	None	agglom15	none	RandomForest	18.1	2.8	0.32	0.15
clear	9	MSC + 1st Der	None	agglom15	none	RandomForest	18.2	2.8	0.32	0.16
clear	10	SNV + 2nd Der	None	agglom15	none	ExtraTrees	18.2	3.3	0.31	0.21
clear	11	SNV + 2nd Der	None	agglom15	none	RandomForest	18.2	2.7	0.32	0.13
clear	12	SNV + 1st Der	None	agglom15	none	ExtraTrees	18.2	2.9	0.32	0.15
clear	13	MSC + 1st Der	None	agglom15	none	LightGBM	18.3	3.1	0.31	0.18
clear	14	MSC + 1st Der	None	agglom15	none	XGBoost	18.5	3.5	0.30	0.16
clear	15	SNV + 1st Der	None	agglom15	none	LightGBM	18.5	4.5	0.30	0.23
clear	16	MSC + 2nd Der	None	agglom15	none	LightGBM	18.7	2.3	0.28	0.10
clear	17	MSC + 2nd Der	None	agglom15	log1p	HistGradientBoosting	18.8	4.9	0.28	0.23
clear	18	MSC + 2nd Der	None	agglom15	none	XGBoost	18.8	3.3	0.27	0.17
clear	19	SNV + 1st Der	None	agglom15	none	HistGradientBoosting	19.0	3.9	0.27	0.18
clear	20	SNV + 2nd Der	None	agglom15	log1p	HistGradientBoosting	19.0	4.3	0.27	0.19
white	1	MSC + 2nd Der	None	median	log1p	LightGBM	15.3	2.0	0.76	0.05
white	2	SNV + 2nd Der	None	median	log1p	LightGBM	16.5	3.5	0.73	0.05
white	3	SNV + 2nd Der	None	median	none	XGBoost	16.6	4.2	0.73	0.05
white	4	SNV + 1st Der	None	median	log1p	GradientBoosting	16.7	2.7	0.70	0.15
white	5	SNV + 1st Der	None	median	none	RandomForest	16.8	5.4	0.72	0.10
white	6	MSC + 2nd Der	None	median	log1p	XGBoost	17.0	4.3	0.71	0.09
white	7	MSC + 1st Der	None	median	none	RandomForest	17.2	4.8	0.70	0.09
white	8	MSC + 1st Der	None	median	none	LightGBM	17.2	5.7	0.70	0.12
white	9	SNV + 2nd Der	None	median	log1p	XGBoost	17.2	3.2	0.70	0.04
white	10	SNV + 2nd Der	None	median	log1p	GradientBoosting	17.4	2.5	0.69	0.06
white	11	MSC + 2nd Der	None	median	none	GradientBoosting	17.5	5.2	0.69	0.11
white	12	SNV + 2nd Der	None	median	none	RandomForest	17.7	5.7	0.69	0.10
white	13	SNV + 1st Der	None	median	none	ExtraTrees	17.8	5.7	0.69	0.11
white	14	MSC + 1st Der	None	median	none	XGBoost	17.8	1.9	0.66	0.15
white	15	MSC + 1st Der	None	median	log1p	LightGBM	17.8	6.6	0.68	0.14
white	16	MSC + 1st Der	None	median	log1p	XGBoost	17.9	5.8	0.68	0.12
white	17	MSC + 2nd Der	None	median	none	LightGBM	17.9	4.8	0.67	0.14
white	18	MSC + 1st Der	None	median	none	ExtraTrees	18.0	5.2	0.68	0.09
white	19	MSC + 2nd Der	None	median	none	HistGradientBoosting	18.1	5.5	0.67	0.14
white	20	MSC + 2nd Der	None	median	log1p	GradientBoosting	18.3	3.9	0.67	0.06
